# Risk factors for mortality in patients with primary biliary cholangitis: a nomogram to predict 5-year survival

**DOI:** 10.3389/fgstr.2025.1534145

**Published:** 2025-03-12

**Authors:** Yaxing Liu, Muyu Gao, Bin Li, Long Liu, Yao Liu, Ying Feng, Xiaojing Wang, Xianbo Wang, Guiqin Zhou

**Affiliations:** ^1^ Division of Comprehensive Internal Medicine, Chifeng Hospital of Traditional Chinese and Mongolian Medicine, Chifeng, China; ^2^ First Clinical Medical College, Beijing University of Chinese Medicine, Beijing, China; ^3^ Center of Integrative Medicine, Beijing Ditan Hospital, Capital Medical University, Beijing, China

**Keywords:** primary biliary cholangitis, age, bilirubin, neutrophil to lymphocyte ratio, risks factor

## Abstract

**Aim:**

The issue of transplant-free survival rate (OS) among patients with primary biliary cholangitis (PBC) remains a persistent concern. In predicting the long-term OS of PBC patients, given the complexity and population specificity of models such as the GLOBE and UK-PBC, our objective is to calculate and assess the risk factors for mortality and 5-year OS among PBC patients based on routine clinical data, ultimately facilitating its clinical application.

**Methods:**

This study enrolled 315 patients with PBC from Beijing Ditan Hospital and randomly divided them into a training cohort (n = 189) and a validation cohort (n = 126). Through Cox regression analyses, we identified risk predictors of mortality to develop a 5-year survival nomogram for PBC. The model was evaluated with Receiver Operating Characteristic (ROC) curves, calibration curves, Decision Curve Analysis (DCA).Kaplan-Meier (KM) curves compared OS across risk groups. Additionally, correlations among the indicators were analyzed.

**Results:**

Ultimately, we established a nomogram incorporating Age, NLR, and TBIL. The Area Under the ROC Curve(AUC-ROC) values for the training and validation groups were 0.7251 and 0.7721, respectively, indicating solid consistency and outperforming the GLOBE model. Calibration and DCA curves further underscored the clinical utility of our model.KM curves revealed the model could differentiate OS across risk levels in subgroup. Additionally, a significant correlation between NLR and TBIL (P=0.0021) was observed, potentially impacting patient prognosis.

**Conclusion:**

We have constructed a well-performing prognostic model based on Age, NLR, and TBIL. This model shows good discrimination, consistency, and clinical use. It helps identifying high-risk patients, enabling more frequent follow-ups and tailored interventions, potentially enhancing prognosis and clinical outcomes.

## Introduction

Primary biliary cholangitis (PBC), also known as primary biliary cirrhosis, is an immune-mediated, chronic progressive cholestatic liver disease with unclear etiology, likely associated with environmental and genetic factors (1).It is defined by non-purulent damage to the small bile ducts (2).The incidence rate in Europe and Asia is 1.9-40.2/100000 and 4.8-5.6/100000, respectively, with a prevalence in middle-aged and elderly women (3–5). Without effective treatment, patients often progress to liver fibrosis, cirrhosis and liver cancer, eventually leading to death (6).

Ursodeoxycholic acid (UDCA) remains the only primary treatment option for PBC. Research indicates that after 5 years of UDCA therapy, the incidence of liver decompensation in PBC patients is between 3.81% and 4.31%, while the rate of hepatocellular carcinoma development is 1.62%. Prognosis worsens notably once cirrhosis develops, with a 5-year survival rate (without the need for liver transplantation) of 77.1% in patients with compensated cirrhosis, dropping to 35.9% in those with decompensated cirrhosis (7).

We conducted a retrospective analysis of clinical data from 315 PBC patients, focusing on liver-related mortality as the outcome, to explore the factors affecting survival. Utilizing the identified risk factors, nomograms were developed to estimate 5-year OS for these patients.

## Materials and methods

### Study population

We accessed the electronic medical record database of Beijing Ditan Hospital and identified 864 patients (n=864) who were hospitalized with PBC from August 2008 to November 2019. After excluding 549 patients based on specific criteria, 315 patients (n=315) remained eligible for inclusion in the study. Case selection was conducted using defined inclusion and exclusion criteria.

Inclusion criteria: 1. Discharge diagnosis of PBC; 2. Each participant received consistent UDCA treatment at a dosage of 13-15 mg/kg daily; 3. Complete follow-up records were available. Exclusion criteria: Patients with comorbid liver diseases (e.g., autoimmune hepatitis, viral hepatitis, drug-induced liver injury, alcoholic liver disease, or liver cancer), those in combination with other second-line therapies (e.g., Obeticholic Acid or Fibrates), those post-TIPS procedure, or post-liver transplantation were excluded. Patients were followed for five years, with liver-related mortality as the endpoint event. PBC was diagnosed according to international guidelines (8), requiring two or more of the following criteria: (i) biochemical markers of cholestasis, such as elevated alkaline phosphatase (ALP); (ii) the presence of anti-mitochondrial antibodies; and (iii) liver biopsy findings consistent with PBC.

### Study variables

Patient demographics, medical history, and laboratory parameters were collected from the hospital’s laboratory department. These included blood counts, such as White Blood Cell Count (WBC), Neutrophil Percentage (NE#), Lymphocyte Percentage (LY#), Platelet Count (PLT), and Hemoglobin (HGB), as well as liver function tests like Alanine Aminotransferase (ALT), Aspartate Aminotransferase (AST), Total Bilirubin (TBIL), Total Bile Acids (TBA), Albumin (ALB), Gamma-Glutamyl Transferase (GGT), and Alkaline Phosphatase (ALP). Additionally, coagulation parameters such as Prothrombin Time (PT), Prothrombin Activity (PTA), and International Normalized Ratio (INR) were evaluated. Immunoglobulin levels, including Immunoglobulin A (IgA), Immunoglobulin M (IgM), and Immunoglobulin G (IgG), were also assessed. Furthermore, specific scores were calculated, such as the Albumin-Bilirubin (ALBI) score, the Mayo risk score, the Aspartate Aminotransferase-to-Platelet Ratio Index (APRI), and the Fibrosis-4 (FIB-4) index. Biochemical response to treatment at 6 and 12 months was also recorded according to the Barcelona Criteria.

### Definitions

The biochemical response to UDCA therapy was determined according to the Barcelona criteria, which specify either a 40% reduction in ALP levels from baseline or a normalization of ALP levels after one year of treatment. In this study, biochemical responses were assessed at both the 6-month and 12-month marks. Liver-related death within 5 years was considered the endpoint. All patients were followed up by telephone until August 2022, with a minimum follow-up period of three years. Patients who survived but were followed up for less than five years were marked as censored. OS was analyzed using the Kaplan-Meier method.

### Statistical analysis

Statistical analyses were executed using R software (version 4.2.0). For data that adhered to a normal distribution, results are reported as mean ± standard deviation, and the independent sample t-test was utilized for comparisons. In contrast, data without normal distribution were represented by the median and interquartile range [M (P25~P75)] and analyzed through the Mann-Whitney U test. Categorical variables were examined using either the chi-square (X²) test or Fisher’s exact test. To pinpoint independent mortality risk factors among PBC patients, univariate and multivariate Cox regression analyses were conducted. A nomogram was developed using R, with validation of the model performed via calibration plots, DCA, and ROC curves. A specific cutoff score was applied to distinguish low-risk and high-risk patient groups, with survival outcomes analyzed using K-M curves.

## Result

### Baseline characteristics

Finally, 315 patients were included. The ratio of females to males is 263(83%) vs 52(17%). The patients were randomly assigned to either a training set (comprising 189 individuals) or a validation set (comprising 126 individuals) in a 6:4 ratio. Statistical analysis revealed no significant differences in baseline characteristics between the two groups, confirming their comparability (**Table 1** **).**

**Table 1:** Baseline demographics, clinical and laboratory characteristics.

	Entire cohort (n = 315)	Training cohort (n=189)	Validation cohort (n=126)	p value
General Features				
Age				0.945
<58	153 (49%)	91 (48%)	62 (49%)	
≥58	162 (51%)	98 (52%)	64 (51%)	
Sex				0.403
Female	263 (83%)	161 (85%)	102 (81%)	
Male	52 (17%)	328(15%)	24 (19%)	
Laboratory indicators				
WBC(10^9/L)				1.000
<4	303 (96%)	182 (96%)	121 (96%)	
≥4	12 (4%)	7 (4%)	5 (4%)	
NLR				1.000
<2	175 (56%)	105 (56%)	70 (56%)	
≥2	140 (44%)	84 (44%)	56 (44%)	
PLT(10^9/L)				0.369
<100	164 (52%)	94 (50%)	70 (56%)	
≥100	151 (48%)	95 (50%)	56 (44%)	
ALT(U/L)				0.175
<40	156 (50%)	100 (53%)	56 (44%)	
≥40	159 (50%)	89 (47%)	70 (56%)	
AST(U/L)				0.098
<35	74 (23%)	51 (27%)	23 (18%)	
≥35	241 (77%)	138 (73%)	103 (82%)	
TBIL(μmol/L)				0.529
<188	132 (42%)	76 (40%)	56 (44%)	
≥188	183 (58%)	113 (60%)	70 (56%)	
ALP(U/L)				0.184
<135	133 (42%)	86 (46%)	47 (37%)	
≥135	182 (58%)	103 (54%)	79 (63%)	
ALB (g/L)				0.670
≥28	65 (21%)	37 (20%)	28 (22%)	
<28	250 (79%)	152 (80%)	98 (78%)	
GGT(U/L)				0.154
<45	61 (19%)	42 (22%)	19 (15%)	
≥45	254 (81%)	147 (78%)	107 (85%)	
TBA(μmol/L)				0.637
<10	50 (16%)	32 (17%)	18 (14%)	
≥10	265 (84%)	157 (83%)	108 (86%)	
IgA(g/L)				0.787
<4.53	259 (82%)	154 (81%)	105 (83%)	
≥4.53	56 (18%)	35 (19%)	21 (17%)	
IgM(g/L)				0.871
<2.74	178 (57%)	108 (57%)	70 (56%)	
≥2.74	137 (43%)	81 (43%)	56 (44%)	
IgG(g/L)				0.561
<15.6	135 (43%)	84 (44%)	51 (40%)	
≥15.6	180 (57%)	105 (56%)	75 (60%)	
PT(s)				0.136
<15.5	290 (92%)	170 (90%)	120 (95%)	
≥15.5	25 (8%)	19 (10%)	6 (5%)	
PTA(%)				0.241
<70	251 (80%)	146 (77%)	105 (83%)	
≥70	64 (20%)	43 (23%)	21 (17%)	
INR				
<1.2	227 (72%)	129 (68%)	98 (78%)	0.086
≥1.2	88 (28%)	60 (32%)	28 (22%)	
Others				
Meld-score	-1.98 (-4.78-1.51)	-2.07 (-4.75-1.70)	-1.98 (-4.81-1.37)	0.574
Child-Pugh				0.300
A	253 (80%)	147 (78%)	106 (84%)	
B	61 (19%)	41 (22%)	20 (16%)	
C	1 (0%)	1 (1%)	0 (0%)	
ALBI	-2.01 (-2.47–1.52)	-2.01 (-2.42–1.52)	-2.09 (-2.52–1.58)	0.304
APRI	1.65 (0.94-2.98)	1.61 (0.91-2.86)	1.65 (0.98-3.18)	0.797
FIB.4	5.7 (2.71-8.94)	5.88 (2.83-9.47)	5.39 (2.6-8.36)	0.440

Over the course of the 5-year follow-up, 28 patients passed away. According to the Barcelona criteria, 60% (188 patients) achieved a biochemical response at 6 months, while 40% (127 patients) did not. At 12 months, the response rate increased to 63% (197 patients), with a non-response rate of 37% (118 patients) (**Table 2** **).**

**Table 2:** Response and death.

	Entire cohort (n = 315)	Training cohort (n=189)	Validation cohort (n=126)
death(5 years)			
No	287 (91%)	170 (90%)	117 (93%)
Yes	28 (9%)	19 (10%)	9 (7%)
death.time(month)	58 (42.5, 83)	59 (45-86)	57 (41-81.75)
Barcelona (6 months)			
No	127 (40%)	70 (37%)	57 (45%)
Yes	188 (60%)	119 (63%)	69 (55%)
Barcelona (12 months)			
No	118 (37%)	65 (34%)	53 (42%)
Yes	197 (63%)	124 (66%)	73 (58%)

### Cox proportional hazards regression

The cut-off value of Age in the training cohort was 57.5, classified dichotomously by 58 years. Albumin levels were stratified using a threshold of <28 g/L in Child-Pugh classification. TBIL levels were dichotomized according to the Child-Pugh and the Common Terminology Criteria for Adverse Events (CTCAE) grading, defined as greater than 10 times the upper limit of normal (ULN). NLR at >2 (9), and PT at >3s above the normal range. All other markers were categorized using laboratory reference ranges as cutoff values. Continuous variables were transformed into categorical variables to enhance model objectivity and simplicity. Through Cox univariate regression analysis of clinical indicators, Age, Gender, NLR, and TBIL were identified as having a significant correlation with the 5-year OS of patients with PBC. In the Cox multivariate analysis, Age, NLR, and TBIL emerged as independent risk factors impacting the 5-year prognosis of these patients (**Table 3**). Their HR and 95% CI were Age:3.56 (1.175-10.805, p=0.025), NLR:3.41 (1.225-9.483, p=0.019), and TBIL:4.99 (1.130-22.023,p=0.034).

**Table 3:** Univariate multivariate analysis of 5-year survival rate in Training cohort.

Factors	Univariable			Multivariable		
	HR	CI95%	p value	HR	CI95%	p value
Age(≥58)	3.668	1.217-11.052	**0.021**	3.56	1.175-10.805	**0.025**
Sex (female)	2.920	1.109-7.692	**0.030**			
WBC (≥4 x 10^9/L)	3.264	0.753-14.135	0.114			
NLR (≥2)	3.827	1.378-10.628	**0.010**	3.41	1.225-9.483	**0.019**
PLT (≥ (10^9/L)	1.373	0.552-3.413	0.495			
ALT (≥40U/L)	1.055	0.429-2.597	0.907			
AST (≥35U/L)	2.036	0.593-6.987	0.259			
TBIL (≥188umol/L)	5.409	1.247-23.465	**0.024**	4.99	1.130-22.023	**0.034**
ALB (<28g/L)	2.512	0.954-6.611	0.062			
ALP (≥135U/L)	2.293	0.826-6.369	0.111			
GGT (≥45U/L)	1.067	0.354-3.216	0.908			
TBA (≥10umol/L)	3.917	0.523-29.348	0.184			
IgA (≥4.53g/L)	1.105	0.367-3.331	0.859			
IgG (≥15.6g/L)	0.902	0.366-2.220	0.822			
IgM (≥2.74g/L)	0.908	0.365-2.257	0.835			
PT (≥15.5s)	0.494	0.066-3.702	0.493			
PTA (<70%)	2.099	0.826-5.332	0.119			
INR (≥2)	1.292	0.508-3.281	0.591			

In univariate analysis, Age, Sex, NLR, and TBIL were predictive factors for the 5-year survival rate in PBC patients. In multivariate analysis, Age, NLR, and TBIL remained significant predictors of 5-year survival. Bold values denote P < 0.05, indicating statistically significant differences.

### Construction and evaluation of the nomogram

**Figure 1:**
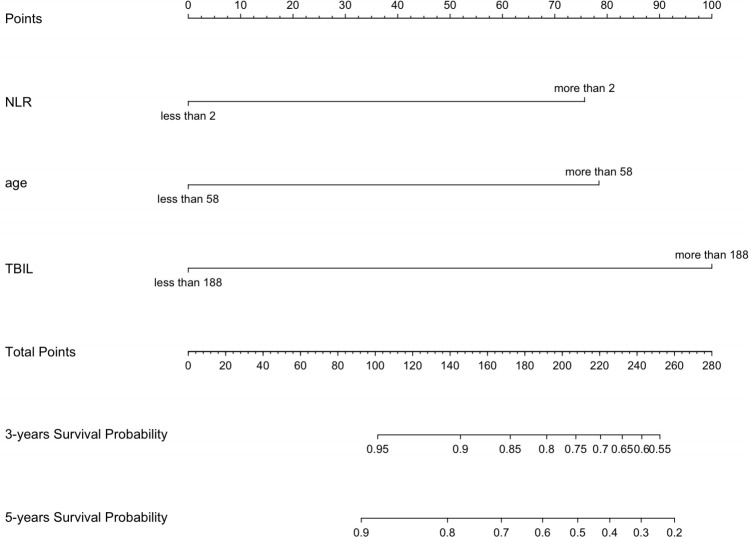
A nomogram developed to forecast the 3-year and 5-year survival rates of PBC patients, utilizing major risk factors influencing mortality.

**Figure 2:**
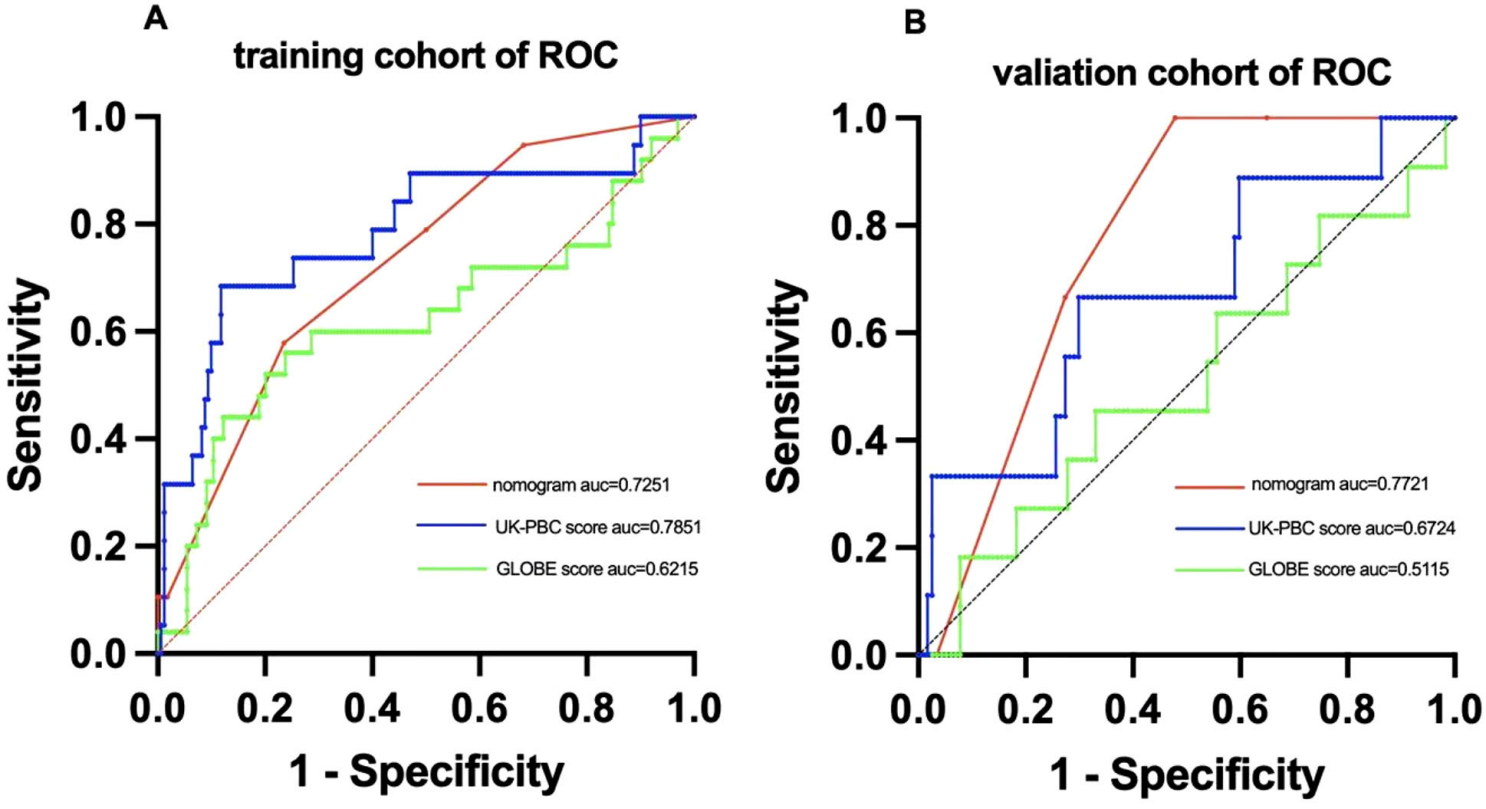
ROC curve analysis comparing the predictive performance of the nomogram,UK-PBC and GLOBE scores in **(A)** the training cohort and **(B)** the validation cohort.

**Figure 3:**
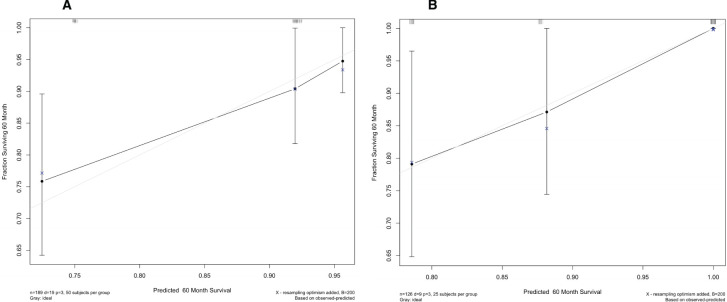
Calibration plots for the nomogram, showing alignment between predicted and actual survival outcomes in **(A)** the training set and **(B)** the validation set.

**Figure 4:**
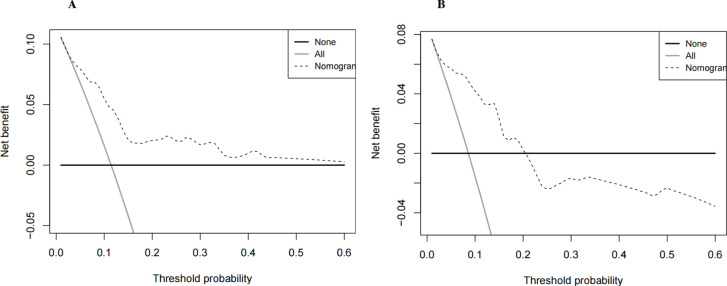
DCA of the nomogram, assessing its clinical utility and net benefit in **(A)** the training set and **(B)** the validation set.

Using multivariate Cox regression analysis, a nomogram based on Age, NLR, and TBIL was created to estimate 5-year survival in PBC patients (**Figure 1**). This nomogram achieved an AUC of 0.7251 (95% CI: 0.6105-0.8396) within the modeling cohort, outperforming the GLOBE score’s AUC of 0.6215 and underachieving the UK-PBC score’s AUC of 0.7851.In the validation group, the nomogram was an AUC of 0.7721 (95%CI:0.6711-0.8730),above GLOBE score’s AUC of 0.5115 and UK-PBC score’s AUC of 0.6724 (**Figure 2**). The calibration curves for both cohorts demonstrated a strong agreement between the predicted and observed 5-year OS (**Figure 3**). DCA demonstrated a strong net benefit for the training group at thresholds of 0.05-0.6 (**Figure 4A**) and in the validation group at thresholds of 0.05-0.2 (**Figure 4B**).

### Low-high risk group for death within 5 years

**Figure 5:**
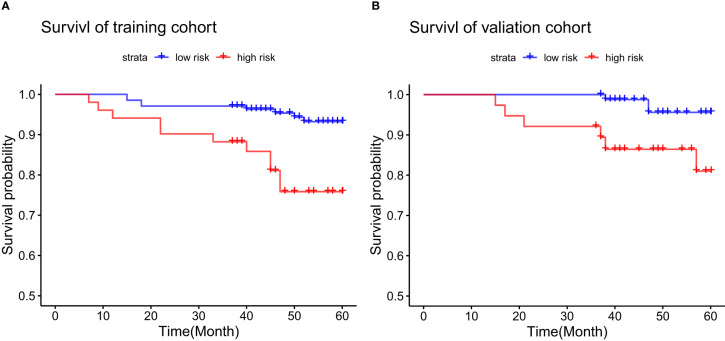
Kaplan-Meier curves comparing 5-year OS between high-risk and low-risk groups in **(A)** the training set and **(B)** the validation set.

Based on a nomogram score cutoff of 116.3, patients were categorized into low- and high-risk categories. In the training cohort, the 5-year OS rate was 93.2% for the low-risk group and 75.9% for the high-risk group, reflecting a significant difference (p<0.001) (**Figure 5A**). For the validation cohort, the low-risk group had a 5-year OS rate of 95.6%, compared to 81.0% in the high-risk group, with statistical significance (p=0.009) (**Figure 5B**).

### Risk factors associated with OS

**Figure 6:**
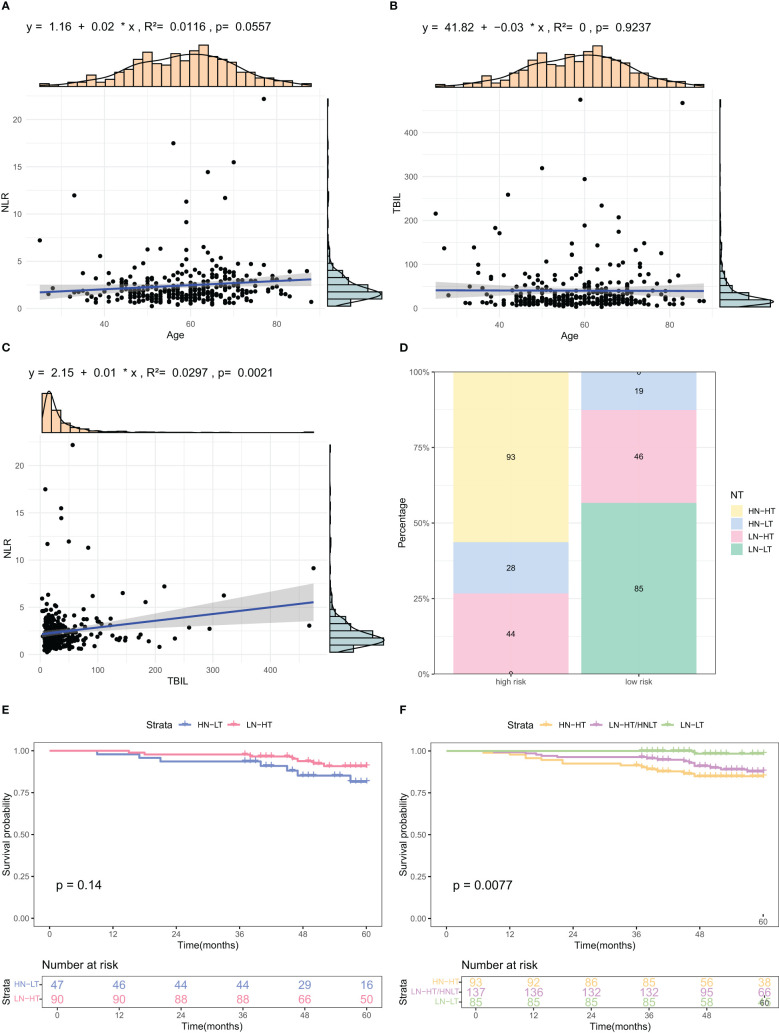
Analysis of relationships among factors included in the nomogram. **(A–C)** Scatter plots illustrating correlations among Age, TBIL, and NLR, the three identified risk factors. **(D)** Stacked bar charts depicting the distribution and percentage of NT indices across different risk categories. **(E, F)** K-M OS curves for subgroups based on combined NLR and TBIL levels. HN-NT (High NLR and High TBIL), HN-LT (High NLR and Low TBIL), LN-HT (Low NLR and High TBIL), and LN-LT (Low NLR and Low TBIL).

Scatter plots revealed a significant correlation between NLR and TBIL (P=0.0021). No significant correlation was found between Age and NLR (P=0.0557) or Age and TBIL (P=0.9237) (**Figures 6A-C**). Binary categorization of NLR and TBIL formed a four-category variable “NT”. The stacked bar chart revealed that the combined variable “NT” was closely associated with low and high risks. The high-risk group had 56% of patients with High NLR and High TBIL (HN-HT), 17% with High NLR and Low TBIL (HN-LT), and 27% with Low NLR and High TBIL (LN-HT), with no patients in the Low NLR and Low TBIL (LN-LT) category. In contrast, the low-risk group had 13% of patients with HN-LT, 30% with LN-HT, and 57% with LN-LT, with no patients in the HN-HT category (**Figure 6D**). Kaplan-Meier curves revealed no significant survival difference between HN-LT and LN-HT groups. Further KM curve analysis confirmed that LN-LT group had the best prognosis, followed by LN-HT/HN-LT group, while the HN-HT group had the poorest prognosis.(**Figures 6E, F** **).**

### Differences in low and high risk groups under Barcelona criteria

**Figure 7:**
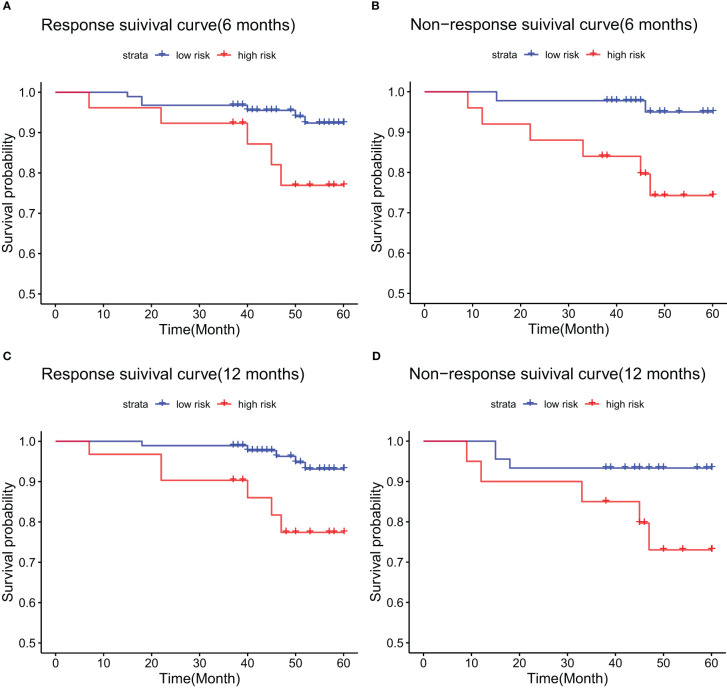
Kaplan-Meier survival curves in the training cohort, stratified by biochemical response to UDCA treatment: **(A)** responders at 6 months; **(B)** non-responders at 6 months; **(C)** responders at 12 months; **(D)** non-responders at 12 months.

Applying the Barcelona criteria to evaluate patients’ biochemical response to UDCA therapy, we examined survival differences between high- and low-risk groups at 6- and 12-month intervals within the modeling cohort. K-M curves demonstrated that among patients showing a biochemical response at 6 months, the OS was 92.6% in the low-risk group compared to 76.9% in the high-risk group, a difference that was statistically significant (p = 0.038) (**Figure 7A**). For patients without a 6-month response, the 5-year OS was 95% for low-risk and 74.3% for high-risk individuals (p = 0.013) (**Figure 7B**). At the 12-month mark, survival rates for responders were 93.1% in the low-risk group versus 77.4% in the high-risk group (p = 0.011) (**Figure 7C**), while for non-responders, the rates were 93.3% in the low-risk group and 73.0% in the high-risk group (p = 0.038) (**Figure 7D**).

**Figure 8:**
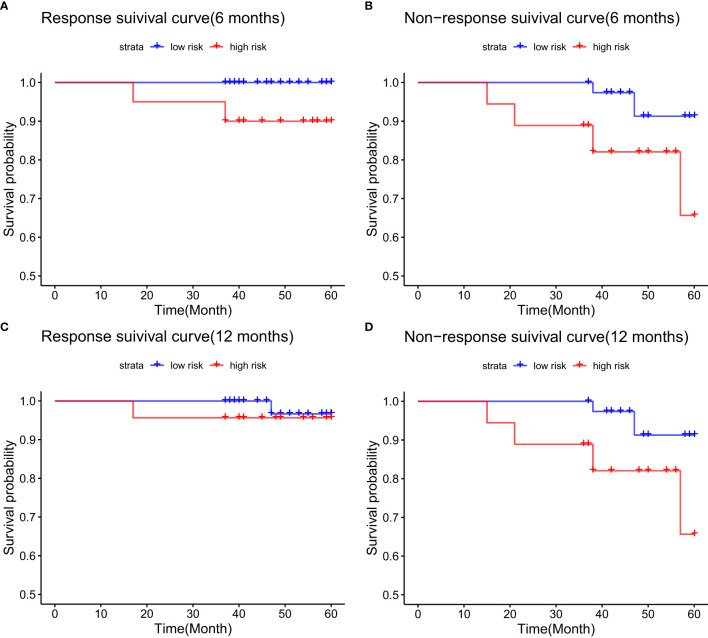
Kaplan-Meier survival analysis for the validation cohort, grouped by biochemical response to UDCA therapy: **(A)** 6-month responders; **(B)** 6-month non-responders; **(C)** 12-month responders; **(D)** 12-month non-responders.

In the validation cohort, patients in the 6-month biochemical response group showed a survival rate of 100.0% for low-risk and 90.0% for high-risk patients, a statistically significant difference (p = 0.025) (**Figure 8A**). For those without a 6-month response, the 5-year survival was 91.3% in the low-risk group compared to 65.6% in the high-risk group, also significant (p = 0.044) (**Figure 8B**). At the 12-month response mark, OS were 96.7% in low-risk and 95.7% in high-risk groups, with no statistical significance (p = 0.612) (**Figure 8C**). In contrast, among patients without a 12-month response, the 5-year survival was 94.3% in the low-risk group and 54.2% in the high-risk group, showing a significant difference (p = 0.002) (**Figure 8D**).

## Discussion

This study included 315 PBC patients, analyzing death as the endpoint to identify risk factors for mortality. Cox univariate regression analysis showed that Age, Sex, TBIL, and NLR significantly influenced mortality risk in PBC patients, while multivariate Cox regression confirmed Age, TBIL, and NLR as independent predictors of death.

As individuals age, the immune system undergoes progressive decline, characterized by diminished functionality of T and B cells, reduced immune tolerance, and elevated levels of pro-inflammatory cytokines. This state of immunosenescence exacerbates autoimmune responses and promotes disease progression in PBC (10, 11). Additionally, the regenerative capacity of hepatocytes in elderly patients is significantly impaired, leading to diminished liver repair mechanisms and accelerated progression of fibrosis and cirrhosis (12). Furthermore, older patients often present with multiple comorbidities which may worsen the severity of PBC, complicate treatment, and increase the risk of mortality (13).Mei Lu (14) et al. observed 3,488 patients with PBC across different ethnicities, categorizing their ages into ≤40 years,41-50 years,51-60 years,61-70 years and >70 years. The prevalence of PBC was found to increase from 3% to 7.5%.Nikolaos K. Gatselis et al. (15) conducted a retrospective study using a database from a prospective study, involving 482 patients with PBC, and found that advanced age was a poor prognostic factor for liver cirrhosis. Similarly, a study conducted in Hong Kong demonstrated that as age increased, the prevalence of PBC rose, and age was found to be a risk factor for overall mortality (HR 1.03, 95% CI: 1.01–1.04) and non-transplant mortality (HR 1.01, 95% CI: 1.0002–1.02) (16). In our study, ROC curves constructed in the modeling group using Prism indicated a cutoff value of 57.5, with patients over 58 showing an increased risk of mortality. This finding aligns with previous reports (17).

The Neutrophil-to-Lymphocyte Ratio (NLR), calculated as the ratio of absolute neutrophil count to absolute lymphocyte count, serves as an indicator of systemic inflammation. Elevated NLR levels suggest an intensified systemic inflammatory response. In the context of PBC, prolonged inflammation may contribute to liver cell injury and exacerbate fibrotic progression (18). Furthermore, a high NLR may reflect suppressed immune function, particularly a decline in lymphocyte-mediated anti-inflammatory and immune-regulatory activities, thereby accelerating disease progression (19). In advanced stages of PBC, infections are commonly observed, and an elevated NLR may signal either an ongoing infection or a worsening inflammatory response, both of which can negatively impact prognosis (20).A high NLR, resulting from increased neutrophils or decreased lymphocytes, is linked to poor outcomes in several cancers (21–23). In a study on hepatocellular carcinoma, Cheng-Hsiang Lo et al. (24) found that both continuous (p=0.006) and categorical (p=0.003) NLR values significantly correlated with survival. Patients with an NLR ≥ 2.4 exhibited an OS of 38.2%, compared to a 1-year survival rate of 83.6% in those with an NLR < 2.4 (p < 0.001). NLR also independently predicted liver-related toxicity (p = 0.002). In lung cancer patients, a high NLR was strongly linked to worse prognosis, with an HR of 1.798 (95% CI: 1.284-2.518, p=0.001) (25). Elevated NLR (>2.46) was an independent risk for reduced non-transplant survival, and when combined with UK-PBC and GLOBE scores, it enhanced prognostic accuracy (26). Despite its association with adverse outcomes, NLR is less frequently reported in PBC studies. Here, we categorized patients using an NLR cutoff of 2 based on Qian Wei et al. (9), grouping them into NLR >2 and NLR ≤2. Findings indicated that NLR was a significant mortality risk factor in PBC, likely due to increased infection risk during cirrhotic decompensation.

Total bilirubin (TBIL) is identified as a key prognostic marker for survival in PBC patients (27, 28). In PBC patients, the intrahepatic bile ducts sustain damage, which disrupts normal bile secretion processes. This impairment leads to an elevation in TBIL levels, indicating a decline in the liver’s metabolic and detoxification capabilities. Consequently, there is an accumulation of endogenous toxins, which exacerbates oxidative stress, ultimately resulting in further hepatic damage (29).In a multicenter study of 4,199 PBC patients treated with UDCA, Willem J. Lammers et al. (30) found that both age (HR: 1.05, 95% CI: 1.04-1.06) and serum bilirubin above ULN (HR: 2.56, 95% CI: 2.22-2.95) were risk factors for death. In another study by Willem J. Lammers et al. (31), 1118 of 4845 patients with PBC were found to end up with liver transplantation or death. It was also found that bilirubin levels had a strong relationship with clinical outcome. The 10-year survival rate for PBC patients with normal bilirubin levels was 86% compared to 41% for those with elevated level. Numerous guidelines, response criteria, and risk scores also support the stabilization or reduction of bilirubin levels to lower disease progression risk (32–35). Carla F. Murillo Perez et al. (36)found that Age, TBIL, and ALP were better markers of 1-year survival in PBC patients. They created the “ABA” tool that categorized the patients into three groups. Low-risk patients were defined as those over 50 years of age with bilirubin ≤1x ULN and ALP ≤3x ULN. High-risk patients were those younger than 50 years and with bilirubin >1x ULN and ALP >3x ULN, while all others fell into the medium-risk category. Initial 10-year survival rates were 89% for the low-risk group, 77% for the intermediate group, and 59% for the high-risk group, with respective 1-year survival rates of 86%, 76%, and 40%.All these indicate that serum bilirubin is an indicator for predicting the prognosis of PBC. In this study, TBIL >10x ULN (188 μmol/L) was shown to be a notable predictor for mortality.

The infiltration of inflammatory cells into areas of hepatic necrosis is a characteristic feature of cholestatic liver injury (37), likely caused by cell swelling, apoptosis, membrane integrity disruption, and release of cellular components due to cholestasis. Damaged cells release pro-inflammatory mediators via damage-associated molecular patterns (DAMPs), which subsequently stimulate immune cells, including macrophages, neutrophils, and natural killer cells, triggering an inflammatory cascade (38). Among the variables included in this study, TBIL and NLR were most strongly correlated. We also found that the NT index, a newly created variable based on these two indicators, revealed differing prognoses across subgroups: best for the LN-LT group, followed by the LN-HT/HN-LT group, and worst for the HN-HT group. This finding is consistent with research on inflammatory mechanisms in cholestasis.

The UK-PBC (32) and GLOBE scores (30), while useful, are complex and challenging to calculate clinically. In this study, the AUC values of the GLOBE score were lower than nomograms in both the validation and training cohorts. Although the UK-PBC score had a higher AUC than the nomogram in the modeling group, it was lower in the validation group. This indicated that neither the UK-PBC nor GLOBE scores were as accurate as the nomogram for this patient cohort, making them less favorable for clinical use. The “ABA” tools of Carla F. Murillo Perez et al. (36) were only used to predict the 1-year prognosis and were not effective for the 5-year prognosis. We constructed a nomogram with three objective variables, Age, NLR, and TBIL, to assess the 5-year OS of patients, the nomograms achieved an AUC of 0.7251 in the training cohort and 0.7721 in the validation cohort, indicating good consistency. The higher AUC value in the validation group was more related to the smaller population in the validation cohort after randomization. The predictions of the calibration curve for 5-year survival in the training cohort were in general agreement with the actual observations. The same results were obtained in the validation group. Simultaneously, we used DCA to assess the model’s clinical utility in predicting 5-year survival for PBC patients. DCA showed clinical value in the modeling group, but the validation group had a narrower threshold, suggesting larger studies are needed to validate this model. The accuracy of the model was thus considered accurate. Utilizing a nomogram threshold of 116.3, patients were stratified into low- and high-risk categories. In the training cohort, 5-year OS were 93.2% for low-risk patients and 75.9% for high-risk patients, showing a statistically significant difference (p < 0.05). Likewise, in the validation cohort, the 5-year survival rates were 95.6% for the low-risk group and 81.0% for the high-risk group. Both groups showed statistically significant differences. The high-risk group had no LN-LT cases, while the low-risk group had no HN-HT cases. HN-HT was prevalent in 56% of the high-risk group, and LN-LT in 57% of the low-risk group, suggesting better 5-year survival with LN-LT and poorer with HN-HT.

This study further analyzed patient responses at 6 and 12 months. Prior research has indicated that the biochemical response at 6 months holds similar predictive value to the response at 12 months (39). We examined whether differences existed between low- and high-risk groups across different time points and with identical biochemical responses. In the training cohort, among those with non-response at 6 months based on the Barcelona criteria, the 5-year OS was 95% in the low-risk group and 74.3% in the high. Among those with a biochemical response at 6 months, the 5-year OS was 92.6% for low-risk and 76.9% for high-risk patients. At the 12-month mark, non-responsers had a 5-year OS of 93.3% in the low-risk group and 73% in the high-risk group. In contrast, responders was 93.1% in the low and 77.4% in the high-risk group.

In the validation cohort, non-responders at 6 months had 5-year OS rates of 91.3% for low-risk and 65.6% for high-risk patients. For patients with a biochemical response at 6 months, the OS reached 100.0% in the low-risk group and 90.0% in the high-risk group. Furthermore, in the poor responder group at 12 months, the 5-year survival rate was 94.3% for low- and 54.2% for high-risk patients. Conversely, within the 12-month responder group, the survival rate was 96.7% for low-risk and 95.7% for high-risk individuals. Significant differences in OS between low- and high-risk groups were observed for response and non-response cases at most time points, except for the 12-month non-response group, likely due to the smaller sample size after stratification.

In the modeling, validation, and different biochemical response groups, excluding the poor responders at 12 months, the low and high-risk groups for PBC patient mortality within 5 years demonstrated statistically significant differences, indicating that the risk stratification for 5-year mortality is meaningful. This study confirms that a patient’s 5-year survival rate can be assessed based on three key indicators: Age, TBIL, and NLR at the time of diagnosis. To further promote the clinical application of this model, we plan to develop a calculation tool (e.g., a mini-program or calculator) based on the nomogram model. This tool will integrate the three core parameters—Age, NLR, and TBIL—to enable rapid risk scoring and classification, while providing intuitive risk levels and survival rate predictions to facilitate clinical decision-making.

Despite the recognized multifactorial nature of mortality determinants in patients with PBC, ALP has been established as an independent prognostic factor in prior studies (40). Though baseline ALP levels between our training and validation cohorts demonstrated uncomparable distributions (P=0.184), multivariate Cox regression analyses did not retain ALP as an independent mortality predictor. Notably, subgroup comparisons of sex-based ALP reference ranges and serial ALP trajectory analyses were not conducted in this investigation. Given the underrepresentation of male patients (n=52, 17%) in our PBC cohort, the methodological constraints preclude robust sex-stratified interpretation. Future work will expand enrollment to evaluate sex-adjusted ALP threshold effects and validate the generalizability of the dynamic stratification strategy based on ULN of ALP across multiple centers. This study has other limitations, as it is based on a single-center analysis with a relatively small sample size. Given that the patients treated at our center often have more severe conditions, there may be inherent biases. To further elucidate the impact of Age, TBIL, and NLR on mortality in PBC patients, we plan to implement regular model updates to continuously optimize its performance. These updates will involve integrating data from multiple regional medical centers to enhance sample diversity and representation, as well as establishing an independent external validation cohort to further evaluate the model’s predictive accuracy and generalizability.

## Main point

1. Age, NLR, and TBIL are independent risk factors for death in PBC patients.
2. The nomogram can predict the 5-year OS.
3. The high-risk group exhibits a shorter OS compared to the low-risk group.

## Data Availability

The raw data supporting the conclusions of this article will be made available by the authors, without undue reservation.
